# Utility of deep learning networks for the generation of artificial cardiac magnetic resonance images in congenital heart disease

**DOI:** 10.1186/s12880-020-00511-1

**Published:** 2020-10-08

**Authors:** Gerhard-Paul Diller, Julius Vahle, Robert Radke, Maria Luisa Benesch Vidal, Alicia Jeanette Fischer, Ulrike M. M. Bauer, Samir Sarikouch, Felix Berger, Philipp Beerbaum, Helmut Baumgartner, Stefan Orwat, for the German Competence Network for Congenital Heart Defects Investigators

**Affiliations:** 1 Department of Cardiology III – Adult Congenital and Valvular Heart Disease, University Hospital Muenster, Albert-Schweitzer Campus 1, Muenster, Germany; 2 Competence Network for Congenital Heart Defects, DZHK (German Centre for Cardiovascular Research), Berlin, Germany; 3 National Register for Congenital Heart Defects, DZHK (German Centre for Cardiovascular Research), Berlin, Germany; 4 Department of Heart-, Thoracic-, Transplantation- and Vascular Surgery, Hannover Medical School, Hannover, Germany; 5 Department of Congenital Heart Disease-Pediatric Cardiology, German Heart Institute Berlin, Augustenburger Platz 1, 13353 Berlin, Germany; 6 DZHK (German Centre for Cardiovascular Research), Partner Site Berlin, Augustenburger Platz 1, 13353 Berlin, Germany; 7 Department of Pediatric Cardiology and Pediatric Intensive Care, Hannover Medical School, Hannover, Germany

## Abstract

**Background:**

Deep learning algorithms are increasingly used for automatic medical imaging analysis and cardiac chamber segmentation. Especially in congenital heart disease, obtaining a sufficient number of training images and data anonymity issues remain of concern.

**Methods:**

Progressive generative adversarial networks (PG-GAN) were trained on cardiac magnetic resonance imaging (MRI) frames from a nationwide prospective study to generate synthetic MRI frames. These synthetic frames were subsequently used to train segmentation networks (U-Net) and the quality of the synthetic training images, as well as the performance of the segmentation network was compared to U-Net-based solutions trained entirely on patient data.

**Results:**

Cardiac MRI data from 303 patients with Tetralogy of Fallot were used for PG-GAN training. Using this model, we generated 100,000 synthetic images with a resolution of 256 × 256 pixels in 4-chamber and 2-chamber views. All synthetic samples were classified as anatomically plausible by human observers. The segmentation performance of the U-Net trained on data from 42 separate patients was statistically significantly better compared to the PG-GAN based training in an external dataset of 50 patients, however, the actual difference in segmentation quality was negligible (< 1% in absolute terms for all models).

**Conclusion:**

We demonstrate the utility of PG-GANs for generating large amounts of realistically looking cardiac MRI images even in rare cardiac conditions. The generated images are not subject to data anonymity and privacy concerns and can be shared freely between institutions. Training supervised deep learning segmentation networks on this synthetic data yielded similar results compared to direct training on original patient data.

## Background

Deep learning technology is currently in the process of revolutionizing medical diagnostic services [1]. Convolutional networks are matching or surpassing human operators in image classification and are increasingly proposed as an adjunct to human medical decision making [2]. Beyond diagnostic classifiers, cardiac chamber segmentation as well as assisted or fully automatic measurement of cardiac function have been developed and are being implemented [3, 4]. Most applications, currently under development require a supervised learning set-up and are thus dependent on labelled medical data for training purposes. While some common disorders should impose virtually no limit on available training material (except for obvious logistic and financial restrictions), in rare medical conditions obtaining an adequate volume of training data may be challenging. Furthermore, in rare disease conditions even pooling actual patient data from multiple institutions may be difficult due to privacy concerns and restrictive local legal regulations. The current project was inspired by the recent development in the field of unsupervised deep learning. Karras and colleagues improved generative adversarial networks (GAN), allowing them to generate naturally looking human faces at a resolution of 1024 × 1024 pixels [5]. Many of the images generated by these novel progressive GANs (PG-GAN) are visually undistinguishable from actual human faces. We adopted this innovative approach to the field of cardiac magnetic resonance imaging (MRI). Specifically, we aimed to test the utility of PG-GANs to generate accurate synthetic equivalents to MRI frames from patients with Tetralogy of Fallot (a form of congenital heart disease in need of regular MRI follow-up). Building on our experience with convolutional segmentation networks we also investigated whether these synthetic images could be used to train downstream deep learning segmentation networks without the need for actual patient data [3, 6].

## Methods

### Overview of the study

**Fig. 1:**
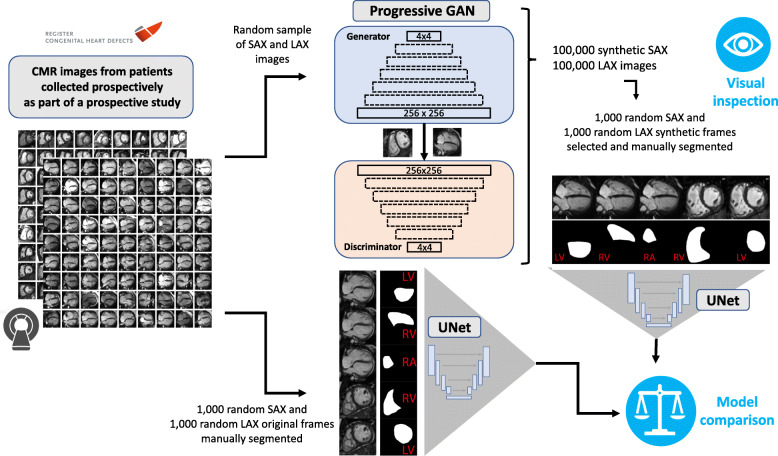
Study overview illustrating the use of original cardiac magnetic resonance (CMR) images for generation of synthetic short axis (SAX) and long axis (LAX) images using a progressive generative adversarial network (PG GAN). The resulting images were subjected to visual inspection by CMR experts and general cardiologists. In addition, deep learning segmentation networks (with U-Net design) were built based, both, on PG GAN and actual CMR frames. The accuracy of the resulting segmentation networks was finally compared on a separate data set not used for training of either network

Fig. 1 illustrates the overall study design. Cardiac MRIs obtained from patients with Tetralogy of Fallot were split into three groups. One part (*n* = 303 patients) was used to train progressive GAN networks, which in turn produced synthetic MRI frames that were utilized to manually produce segmentation maps for the training of downstream U-Net segmentation models. A second part (*n* = 42 patients) was utilized to train U-Nets directly on patient frames. The performance of these two U-Net models was subsequently compared with a third (independent) fraction of the data (*n* = 50 patients), not used for training either U-Net models. In addition, the quality of the synthetic PG-GAN images was assessed visually, and the degree of similarity to original MRI images was quantified using a statistical similarity index (for details see below).

### Progressive GAN (PG-GAN)

To generate realistic images of long and short axis cardiac MRI frames, two progressive GANs were built as described in detail by Karras et al. 2018 [5]. The original network was modified for the specific requirements of our dataset based on the *GANLib* GitHub repository and implemented in TensorFlow [7]. Adaptations compared to the original publication included the reduction of the output dimension to one channel (to account for grayscale MRI frames) and the reduction of the maximum image size to 256 × 256 pixels to account for the available computing power compared to the published commercial NVIDIA setup. As in the original publication, the current PG-GAN was grown progressively, increasing image size from 4 × 4 pixels to 8^2^, 16^2^, 32^2^, 64^2^, 128^2^ and 256^2^ pixels, respectively. The number of filters and the batch size was adjusted accordingly (for details see below). A latent vector of dimension 64 was used as an input to the generator which consisted of blocks of 4 × 4 and 3 × 3 2-D convolution layers with leaky ReLU (leakiness 0.2) and a 2-D upscale layer. In analogy to the original model, new layer-blocks were added to both the generator and the discriminator incrementally, while existing layers remained trainable. Additional layers were faded in, doubling the resolution of the generator and the discriminator but allowing for a smooth transition in the process. The addition of minibatch standard deviation into the discriminator and pixel-wise feature vector normalization in the generator were also implemented as originally described [5]. The corresponding discriminator had a symmetric design with layer blocks of 3 × 3 and 4 × 4 convolutional layers (including leaky ReLU) and a 2-D average pooling layer. Filter number was 48, 32, 24, 16, 16, 16 and 16 respectively for the 3-layer blocks. Adam optimization was employed and the Wasserstein distance served as distance metric [8]. During training, the batch size was decreased as the resolution increased to match available memory constraints from 64 to 16 samples. Training of the model for 124,000 epochs on a Windows i9 PC with an NVIDIA GeForce RTX 2080Ti graphic processing unit required approximately 12 h per model.

### Dataset for PG-GAN training

Overall, 6400 4-chamber long axis (LAX) MRI frames from 279 patients and 7015 2-chamber short axis (SAX) images from 303 patients (57.8% male patients, median age [IQR] 15.0 years [12.8–19.3 years], height 170 cm [163–177 cm], weight 54.0 kg [43.0–69.9 kg]) were used for training the PG-GANs. All patients had a diagnosis of congenital heart disease with a status post repair for tetralogy of Fallot - a form of cyanotic congenital heart disease which accounts for approximately 12% of adults with congenital heart disease under regular follow at specialized centers [9]. The patients formed part of a prospective nationwide study initiated and conducted by the Investigators of the German Competence Network for Congenital Heart Defects between 2003 and 2009 (Follow up of Post- Repair Tetralogy of Fallot; www.ClinicalTrials.gov; unique identifier, NCT00266188). Inclusion criteria were absence of an implantable cardioverter-defibrillator and a patient age at the time of MRI > 8 years. The MRIs were collected at 14 German centers using a pre-defined protocol. Further details on the MRI protocol as well as additional exclusion criteria have been reported by the study consortium previously [10–12]. All MRI cine loops were saved in DICOM format in a centralized digital imaging database. These archived cine loops were made available for the current study. All patients included are enrolled in the National Register and approval of the study protocol was obtained from the appropriate ethics committee. The included subjects gave appropriate informed consent before the baseline MRI investigation and study inclusion.

### Administrative permissions / ethics approval

All study participants (or their legal representatives) gave written informed consent before the baseline MRI investigation and study inclusion, which were approved by the Ethics Committee (Ruhr University Bochum, Bad Oeynhausen, Germany, Reg.-No. 14/03). In addition, research within the framework of the National Register for Congenital Heart Defects is covered by Ethics Approval by the Charité Ethics Committee, Berlin, Germany.

### Visual assessment of the PG-GAN results

To evaluate the quality of the synthetic PG-GAN network frames, a random selection of 200 PG-GAN derived, and 200 original MRI frames were presented to human investigators head to head. The operator was presented with two images in a random order arrangement (one PG-GAN based, one original) and was required to determine which image was of GAN origin. The number of correct answers is reported as a percentage of total pairs presented, representing a measure of the discriminatory ability of human operators. To test whether experienced cardiac MRI specialists may have a superior ability to recognize synthetic images compared to cardiologists not directly involved in cardiac MRI reporting the results were compared using the Fisher exact test and *p*-values are reported.

### Identification of similarities between GAN images and original patient frames

To identify similarities between the generated PG-GAN frames and natural MRI frames available in the dataset a multi-scale statistical similarity index (sliced Wasserstein) distance approach at various resolutions is adapted [5]. To this end, a Laplacian pyramid of the images was created, and the Wasserstein distance was calculated for a series of pixels in both the PG-GAN and all the available original images. The images with the lowest sliced Wasserstein distance were considered to be the most similar to the synthetic PG-GAN frame in question.

### Segmentation network (U-net)

**Fig. 2:**
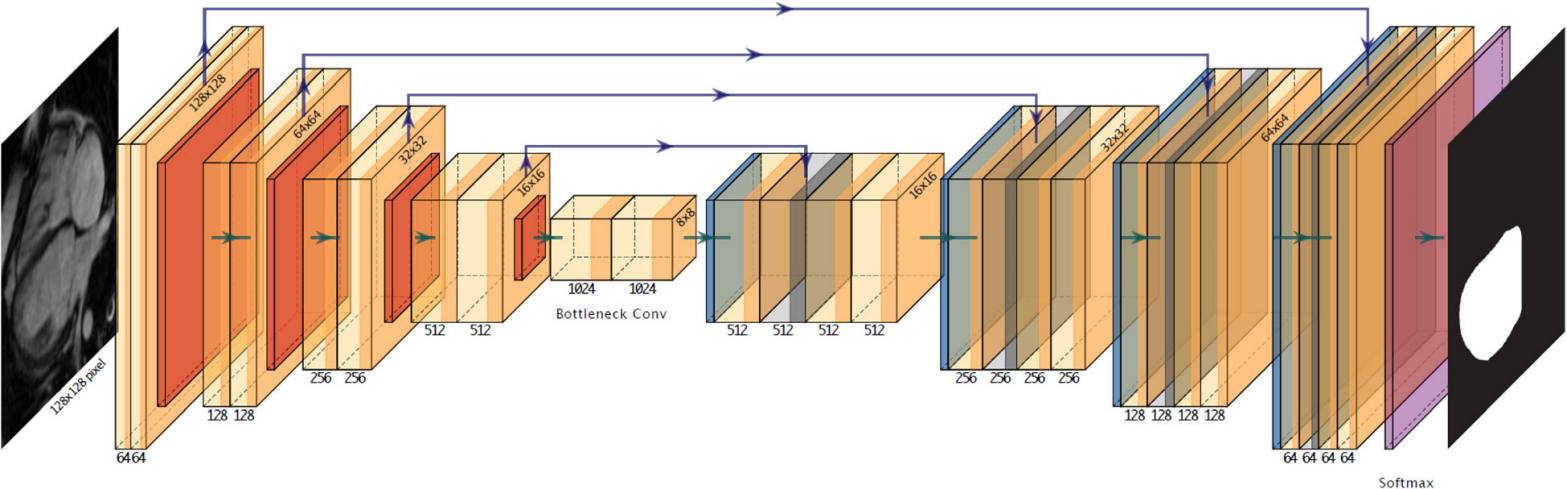
Illustration of the network design of the U-Net segmentation network. The network accepts a greyscale frame (128 × 128 pixels) and produces segmentation maps of equal size for the heart chambers involved. The network consists of a contracting path with multiple 3 × 3 convolutions followed by ReLU (Rectified Linear Unit) activation and a max. Pooling operation (2 × 2). The number of channels is doubled at each step of the contraction path. In the expanding part, the feature maps are upscaled symmetrically, with 2 × 2 up-convolutions. In addition, channels of the expanding path are combined with the corresponding part of the contracting path through concatenation. The number on top corresponds to the number of channels, while the dimensions are given on the left of the respective boxes. For details see Ref. [13]

For segmentation of cardiac chambers, a U-Net setup was employed [13]. The network is illustrated in Fig. 2. It accepts individual MRI images at a resolution of 128 × 128 grayscale pixels´ and returns segmentation maps for the various cardiac chambers (left ventricle [LV], right ventricle [RV] and right atrium [RA]). For training, the model was presented with raw images as well as manually produced masks (RV and LV for the SAX view or RV, LV and RA for the LAX view). Overall, 1000 pairs of original SAX and LAX images with corresponding maps were produced and were the basis of U-Net training. These image/mask pairs were derived from 42 ToF patients not used for PG-GAN training. To increase the heterogeneity of the data image augmentation was applied to all 1000 frames and masks (rotations ±20°, width and height shifts of 5% as well as shears and zoom of up to 20 and 10%, respectively, with horizontal or vertical flipping disabled) resulting in 10,000 augmented image/mask pairs. Training was performed using Intel i7 and i9 computers equipped with NVIDIA GeForce GTX 1070 and GeForce RTX 2080Ti graphic processing units. For training, a validation split of 5% was employed. The U-Net was implemented in R (TensorFlow version 1.8; keras package version 2.1.6; CUDA version 9.0.176) as previously described [3, 13].

In total, two pairs of U-Net models were produced. One pair (including a SAX and a LAX model) based on a training data set using original patient MRI frames and a second pair trained on a random sample of frames produced by the PG-GAN model.

### Comparison of segmentation network (U-net) performance

To assess performance differences between U-Nets trained on synthetic PG-GAN derived data from those trained directly on patient MRI frames, the Dice metric and percentage area variability (ratio of the area difference between actual and predicted area, divided by the actual area) were assessed for both models compared to ground-truth masks produced manually on a set of frames from patients not used for model training. Details on the calculation of Dice metrics and percentage area variability have been reported in detail in the literature by us and others previously [3, 4]. Briefly, the Dice metric assesses the overlap between U-Net derived and the ground-truth segmentation. The value of the metric will bin in the range of 0 to 1, with 0 indicating the worst possible segmentation (no overlap) and 1 corresponding to a perfect segmentation result. Differences for these metrics between the PG-GAN and actual patient MRI-based U-Nets was tested by using (paired) Wilcoxon’s rank sum tests.

## Results

### Feasibility of PG-GAN training and visual results

**Fig. 3:**
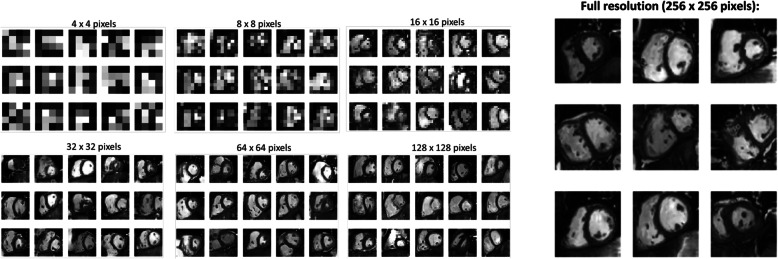
Overview over the training of the progressive adversarial network (PG GAN) using increasing image resolution of 4 × 4, 8 × 8, 16 × 16, 32 × 32, 64 × 64 and 128 × 128 pixels. Finally, a maximal resolution of 256 × 256 pixels is achieved (right panel)

**Fig. 4:**
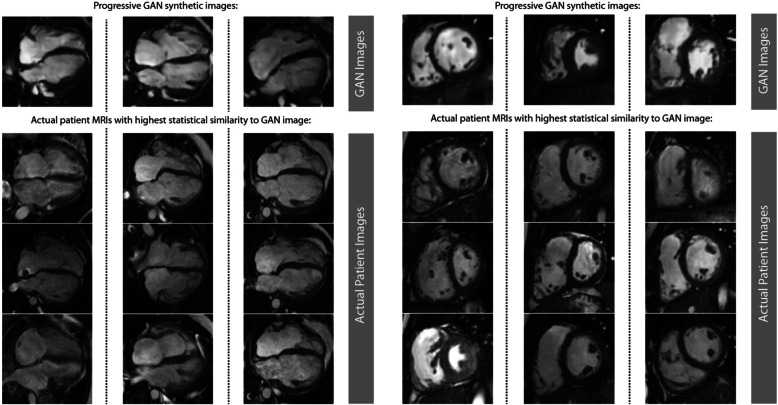
Comparison of synthetic cardiac magnetic resonance (CMR) images (top row) produced by the progressive generative adversarial network (GAN) with actual CMR images from patients with tetralogy of Fallot with the highest degree of statistical similarity (Wasserstein distance; for details see Method section)

The first aim of the study was to test the feasibility of training the PG-GANs on the data available. We found no evidence of training instability in our models. Figure 3 illustrates the progress of image generation as the resolution was increased during training from 4^2^ to 128^2^ and 256^2^ pixels. All GANs trained as expected and yielded visually acceptable synthetic MRI frames. Based on the results of the multiscale statistical similarity between PG-GAN generated frames and actual patient MRI frames, Fig. 4 shows a comparison between three representative PG-GAN generated images (top row), and respective actual patient images with the lowest Wasserstein distance.

Presenting 200 pairs of randomly positioned images (one from the PG-GAN, one original MRI frame) to study subjects with various grades of experience showed that 68.7 and 85.3% of the short axis images generated by the PG GAN were recognized as such by experienced cardiologists (GD, AF and UB) and CMR experts (RR and SO), respectively. For 4-chamber views the correct recognition rate was 72.2% for non-CMR specialists and 88.0% for the experienced CMR readers. The trained and experienced CMR-experts performed significantly better compared to the cardiologists not directly involved in cardiac MRI reporting (*p*-value < 0.001 for both short and long axis frames). Overall, however, none of the PG-GAN derived frames was labelled as anatomically implausible by the reviewers.

### Results of segmentation training based on PG-GAN data

The performance of trained U-Net models was tested on a set of 100 MRI frames from patients not used for PG-GAN or U-Net training and the percentage variation as well as the Dice metric was quantified. Comparing segmentation networks (U-Net) trained on actual patient MRIs and those trained entirely on PG-GAN derived data showed only slight superiority in performance for the former. As shown in Table 1 while U-Nets trained on patient data directly had statistically significantly better results, the actual values were very similar between the models. The absolute difference between the models is less than 1% for comparisons.

**Table 1:** Comparison between the segmentation accuracy

Cardiac Chamber	Pg-GAN	Actual pat. MRI	***p-value***
**Percent Variation**			
**Long axis view:**			
Left Ventricle	0.021 [0.017–0.027]	0.014 [0.012–0.018]	*< 0.0001*
Right Ventricle	0.019 [0.016–0.024]	0.016 [0.012–0.022]	*< 0.0001*
Right Atrium	0.014 [0.011–0.018]	0.011 [0.009–0.014]	*< 0.0001*
**Short axis view:**			
Left Ventricle	0.013 [0.010–0.019]	0.013 [0.010–0.017]	*0.41*
Right Ventricle	0.035 [0.025–0.042]	0.036 [0.028–0.050]	*0.003*
**Dice Metric**			
**Long axis view:**			
Left Ventricle	0.978 [0.973–0.983]	0.986 [0.982–0.988]	*< 0.0001*
Right Ventricle	0.981 [0.976–0.984]	0.984 [0.978–0.988]	*< 0.0001*
Right Atrium	0.986 [0.983–0.989]	0.989 [0.985–0.991]	*< 0.0001*
**Short axis view:**			
Left Ventricle	0.987 [0.982–0.991]	0.987 [0.983–0.990]	*0.45*
Right Ventricle	0.965 [0.958–0.975]	0.964 [0.951–0.972]	*0.002*

Comparison between the segmentation accuracy (percent variation and Dice metric) between U-Net based segmentation models trained entirely on synthetic frames generated by the generative adversarial network (PG GAN) and those trained on actual patient magnetic resonance imaging (MRI) frames. *p*-values were calculated using a paired non-parametric test

## Discussion

The current study demonstrates the use of GANs to generate synthetic cardiac MRI images of patients with congenital heart disease. As data quantity and quality are critical for training deep learning models, the proposed method should be useful to assist training downstream deep learning networks in the setting of rare medical conditions. The synthetic GAN images are not subject to data anonymity issues or privacy concerns and can be shared freely between medical institutions, allowing accelerated development of new diagnostic tools.

Artificial intelligence and deep learning solutions are revolutionizing interpretation of medical images. It is hoped that these technologies will not only augment efficiency but also improve diagnostic quality. Most current implementations use image classifiers or segmentation networks to this end [14, 15]. These technologies accept a high dimensional input (generally an image) and yield a lower dimensional output such as assigning the image to a limited number of possible diagnostic groups or classifying image pixels to particular anatomic segments. The approach presented in the current paper takes the opposite (and arguably more challenging) approach of mapping a low dimensional vector to a realistic, anatomically plausible cardiac MRI image. In 2014 Goodfellow proposed the concept of generative networks to achieve this goal. The GAN network consists of two distinct parts that work in synergy: a generator sub-network takes actual low dimensional (random) vector data and attempts to construct a plausible high-resolution image. In addition, a discriminator is added to distinguish between the synthetic images produced by the generator and real images. These two parts of the model are trained together, thus improving both their generative and discriminatory ability in the process. Despite impressive early results, conventional GANs are inherently difficult to train and suffer from training instability. This is partly explained by the fact that optimizing GANs resembles a prisoner’s dilemma type set-up, where generator and discriminator weight have to be optimized in synergy and are dependent on each other [16]. While these issues are manageable for low resolution images, training GANs becomes increasingly challenging with growing image resolution. Intuitively this appears plausible, as starting with a high-resolution image makes the task of classifying the image as real or synthetic much easier compared to the task of generating a near-accurate image from scratch. Thus, the task of the discriminator is more manageable, and it tends to dominate early in the training process, therefore preventing successful training. The novel approach introduced by Karras et al. was to start with a low-resolution GAN and increasing image size step by step during training (hence the name progressive GAN), thereby supporting the generator and stabilizing the model [5]. In 2017 the group demonstrated the utility of this approach by generating a large number of high resolution (1024 × 1024 pixel) synthetic images of human faces.

Previous applications of GAN models to medical imaging include increasing the resolution of cardiac MRI images [17], de-aliasing images [18] as well as converting imaging appearance from one modality (e.g. CT) to that of another imaging technique (e.g. MRI) [19]. In addition, Shin and colleagues, used conventional GANs to generate synthetic images of brain MRI in patients with Alzheimer disease or brain tumors with a resolution of 128 × 128 pixels [20]. The authors emphasize the potential of the technology to increase training data availability as well as overcome restrictions around data anonymity. To the best of our knowledge, our study is the first to apply progressive GANs to generate realistic cardiac MRI images for patients with congenital heart disease. The resolution achievable with this approach is at the upper end of the published medical literature. Even higher resolution, however, should be possible with improved technology and especially more powerful computing capabilities. The main appeal of synthetic PG-GAN images is the potential to use these anatomically accurate images for training of downstream networks, without anonymity concerns. Not surprisingly, MRI specialists were able to identify most of the synthetic images correctly. However, to the largely untrained eye the images look accurate and this was reflected by the much lower ability of non-specialists to correctly identify synthetic images. In addition, the frames are anatomically accurate and training segmentation networks based on the generated data is feasible. We built on our previous experience with U-Net segmentation deep learning networks and trained these models both on PG-GAN images and actual patient data. While the latter models produced statistically significantly higher Dice scores and lower area variation compared to manual ground-truth masks, the actual difference between the networks is negligible (< 1% in absolute) terms. We, therefore, contend that segmentation networks should be trainable on synthetic GAN images and deliver accurate clinical results. Additional benefits of PG-GAN derived images include the potentially lower cost of obtaining these frames as well as possibility to add anatomic variation or other sources of heterogeneity to the data, potentially benefiting segmentation network training (e.g. by reducing overfitting problems).

### Limitations

We have not investigated whether dynamic series of images mimicking cardiac motion could be generated by adjusting the input vector. It has been reported that manipulating the latent vector can result is meaningful transitions between images. Due to the limited resolution and the fact that visually especially the blood pool is not perfectly modelled by the generator, the images created are partly distinguishable from actual patient frames. It is hoped that by optimizing the GAN network further, increasing computing power and potentially combining the PG-GAN setup with other downstream deep learning networks the image quality can be further improved. We can only speculate on the reasons why no evidence of training instability was evident for the PG-GAN in our study. This may be potentially related to the design of the PG-GAN making it less prone to such effects compared to conventional GAN setups [5].

## Conclusions

The current study illustrates the utility of PG-GANs for generating large amounts of realistically looking cardiac MRI images even in rare cardiac conditions. The generated images are not subject to data anonymity and privacy concerns and can be shared freely between institutions. As training supervised deep learning segmentation networks on this synthetic data yielded similar results compared to direct training on original patient data, we contend that this approach may find applications for training segmentation networks or improving accuracy of existing models by additional training on PG-GAN generated images.

## Data Availability

The datasets used and/or analyzed during the current study are available from the corresponding author on reasonable request.

## Abbreviations

CUDA: Compute Unified Device Architecture
DICOM: Digital Imaging and Communications in Medicine
GAN: generative adversarial networks
LAX: long axis
LV: left ventricle
MRI: magnetic resonance imaging
PG-GAN: progressive generative adversarial networks
RA: right atrium
ReLU: rectified linear unit
RV: right ventricle
SAX: short axis
